# Associations of Body Mass Index and Lifestyle Factors with Suicidal Ideation, Planning, and Attempts Among Korean Adolescents: A Cross-Sectional Study

**DOI:** 10.3390/healthcare13121470

**Published:** 2025-06-18

**Authors:** Haitao Wang, Kyung-O Kim

**Affiliations:** 1 Sociology of Sports Active Aging Laboratory, Kyungpook National University, Daegu 41566, Republic of Korea; 2023034630@knu.ac.kr; 2 Department of Physical Education, Kyungpook National University, Daegu 41566, Republic of Korea

**Keywords:** health behaviors, lifestyle, adolescents, suicide, body mass index, cross-sectional study

## Abstract

Background: Unhealthy lifestyles constitute significant risk factors for adolescent suicide, and their detrimental effects may persist from adolescence into adulthood. This research study sought to examine how Body Mass Index (BMI), alongside various lifestyle behaviors among teenagers in Korea, correlates with suicidal thoughts, the formulation of suicide plans, and actual suicide attempts. Methods: The research examined unprocessed information collected during the 2022 Korean Youth Risk Behavior Web-based Survey (KYRBS), which was administered by the Korea Disease Control and Prevention Agency (KDCA). Lifestyle factors associated with suicidal behavior were selected as independent variables. The sample was stratified according to BMI for further analysis. Logistic regression models were applied to assess the association between lifestyle factors and the risk of adolescent suicide. Results: The analysis identified significant correlations between unhealthy dietary patterns, hazardous drinking behavior, smoking, and a sleep duration of less than 5 h, all of which were associated with a heightened suicide risk among adolescents. Notably, underweight adolescents who had a sleep duration of less than 5 h demonstrated a markedly elevated risk of suicidal ideation (OR = 2.391, 95% CI [1.035–5.525]). Among overweight adolescents, frequent coffee consumption was significantly associated with both suicidal planning (OR = 1.850, 95% CI [1.133–3.020]) and suicide attempts (OR = 1.958, 95% CI [1.024–3.742]). Importantly, hazardous drinking behavior was strongly associated with suicide attempts (OR = 2.277, 95% CI [1.132–4.580]). Non-smoking behavior exhibited a significant relationship with a decreased likelihood of suicidal ideation (OR = 0.706, 95% CI [0.507–0.983]) and suicidal planning (OR = 0.528, 95% CI [0.299–0.930]). Furthermore, among obese adolescents, non-smoking behavior significantly decreased the risk of suicidal ideation compared to smoking (OR = 0.514, 95% CI [0.297–0.887]). Conclusions: The study revealed that the combined impact of unhealthy behaviors—smoking, eating an unhealthy breakfast, sleeping for less than 5 h, and hazardous drinking behavior—significantly affect suicide-related behaviors in adolescents. The interaction between BMI and lifestyle factors is a critical determinant of these behaviors. Specifically, sleep health exerts a substantial influence on suicide-related behaviors in underweight adolescents, while smoking strongly correlates with suicidal behaviors in overweight and obese adolescents. Targeted attention to the interplay of smoking, diet, sleep, and alcohol consumption with BMI is crucial for the early detection and prevention of adolescent suicide.

## 1. Introduction

Self-inflicted death represents a significant worldwide health crisis and currently ranks as the fourth most common cause of mortality for young people across the globe, emphasizing the pressing requirement for preventative strategies [1]. In many countries, the mortality rate from self-harm is significantly underreported due to societal stigma, cultural expectations, and legal constraints, with adolescents constituting a particularly high-risk demographic [2]. In South Korea, the combined suicide rate for children and adolescents aged 10–19 is 3.77 per 100,000, rising to 6.04 per 100,000 among older adolescents aged 15–19 [3]. According to 2021 Korean mortality statistics, 13,352 individuals died by suicide, and the rate of self-harm and suicide among teenagers and young adults had increased by 15.4% over the previous decade [2]. The rising prevalence of self-harm and suicide has increased injury treatment costs to 5.3 trillion KRW, placing additional strain on the healthcare system [4].

From a social-ecological perspective, the formation of adolescent suicidal ideation and behavior results from the interaction of individual, family, peer relationship, school environment, and broader sociocultural factors [5]. Within this social-ecological framework, individual characteristics such as Body Mass Index (BMI) and lifestyle should no longer be understood as isolated biological indicators, but rather embodied manifestations of social processes. Chronic social stress can gradually wear down the body’s biological systems over time [6].

Previous research has shown that BMI has complex associations with suicidal ideation and behaviors [7]. Specifically, male BMI shows a negative linear association with suicide attempts, while non-depressed females show an L-shaped association, indicating an inverted U-shaped trend between BMI and suicide [8,9]. Weight loss during adolescence, leading to being categorized as underweight, has also been linked to suicidal ideation [10]. Adolescent overweight and obesity show positive correlations with suicidal ideation and planning [11,12]. These outcomes are often closely related to social factors such as weight discrimination, body image distress, and social exclusion [13]. Children with excessive obesity are more susceptible to emotional disorders and less likely to engage in health-promoting behaviors [14], which more reflects the challenges they face in peer relationships, social acceptance, and self-identity [15]. Some studies suggest that obese adolescents are more likely to make plans for suicide [16]. Research on adolescent weight perception and mental health revealed that girls who perceived themselves as overweight exhibited more depressive symptoms, with 0.32 standard deviation units in 1986, 0.33 in 2005, and 0.56 in 2015 [17]. These findings collectively underscore the complex bidirectional relationship between weight status, body weight perception, and adolescent mental health, highlighting the critical need for comprehensive interventions that address both actual weight management and body image distortion in this vulnerable population.

On the other hand, a U.S. study identified a link between various lifestyle factors and suicide [18]. Sleep problems are a short-term risk factor for suicidal thinking among high-risk youth [19]. A U.S. cross-sectional study suggested that skipping breakfast for seven consecutive days was linked to suicidal ideation [20]. Adolescents who were sedentary for more than three hours per day were more likely to engage in suicidal behaviors compared to those with less sedentary time [21]. Other modifiable lifestyle factors, such as physical activity [22] and smoking [23], have also been explored for their effects on suicidal behaviors. Inadequate sleep, insufficient physical activity, poor breakfast intake, unhealthy body weight, alcohol consumption, smoking, and sedentary behavior have all been linked to suicide [24,25,26]. These lifestyle patterns reflect broader social and cultural influences rather than isolated individual choices. School environments, community safety, and community resources determine adolescents’ access to nutritious foods, safe recreational spaces, and opportunities to engage in healthy behaviors [27]. The clustering of unhealthy lifestyle behaviors often indicates underlying social stressors rather than simple personal preferences [28]. Changes in both physical and mental health during adolescence are closely tied to lifestyle factors, and adolescence is a critical period that can influence health outcomes into adulthood.

The relationship between lifestyle factors and adolescent suicidal behaviors has not been fully explored, particularly in comprehensive contexts that consider multiple factors simultaneously. While previous studies have examined isolated lifestyle elements, our research adopts a BMI-stratified analytical approach to investigate potential differential effects across weight categories. Through subgroup analyses based on BMI, we aim to understand how weight status influences these associations within individual–environment interactions. This study, utilizing a nationally representative sample from South Korea, comprehensively examines the combined effects of multiple lifestyle factors—such as smoking, dietary habits, physical activity, sedentary behavior, sleep duration, and alcohol consumption—on suicidal ideation, planning, and attempts. Our analytical approach provides insights into how risk patterns may vary across different adolescent weight groups and explores the interaction effects between lifestyle factors and weight status.

We hypothesize the following: (1) Certain lifestyle factors will show stronger associations with suicidal behaviors than others; and (2) These associations will show significant variation across BMI categories.

## 2. Methods

### 2.1. Data Sources and Study Design

**Figure 1:**
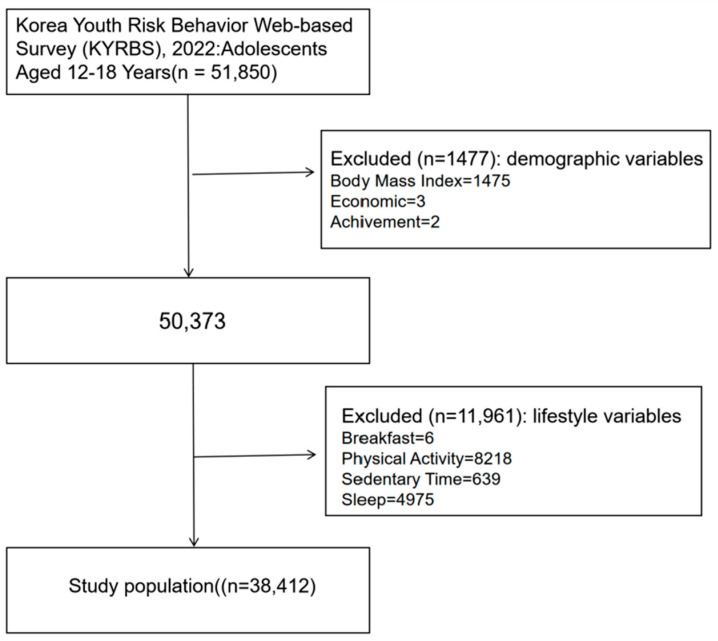
Flow chart for subject identification.

This study used data from the KYRBS conducted by the KDCA. The KYRBS is an ongoing nationwide cross-sectional survey designed to assess health risk behaviors among middle and high school students and to provide data for the development and evaluation of school health policies and programs in Korea. KYRBS employs a multi-stage cluster sampling design to obtain a nationally representative sample of Korean middle and high school students. The population includes all public and private middle and high school students across 17 provinces in Korea. Since 2010, the KYRBS has been conducted annually from June to July. Detailed information about the KYRBS is available at www.kdca.go.kr (accessed on 15 March 2024) and in other publications [29,30]. Since KYRBS data is publicly available, Institutional Review Board (IRB) approval was not required for this study. Nevertheless, the investigator ensured all study subjects were notified about the principles outlined in the Helsinki Declaration. Of the 51,850 individuals surveyed in the 2022 KYRBS, adolescents aged 12–18 were included. This study employed complete case analysis to handle missing data, whereby participants with missing values were excluded from the analysis. A total of 13,438 participants were excluded, accounting for 25.92% of the original sample. Following removal of cases with missing information, the analysis incorporated 38,412 individuals as the final study population (Figure 1).

### 2.2. Dependent Variable

This investigation measured three key dependent variables: thoughts about ending one’s life, development of concrete strategies for suicide, and actual attempts to engage in self-harm during the previous 12 months. The questionnaire posed the following queries: “During the past year, have you seriously contemplated taking your own life?”, “Within the last 12 months, have you formulated a detailed plan to commit suicide?”, and “In the previous year, have you tried to end your life?” Furthermore, suicidal conduct was characterized as experiencing any of these three phenomena within the preceding year, a conceptualization that has been applied in additional research focusing on adult populations and in studies using the KYRBS [18].

### 2.3. Independent Variables

#### 2.3.1. Sleep Time

Following the National Sleep Foundation’s sleep duration guidelines specifically developed for adolescents, this study categorized participants’ sleep into four categories: sleeping for a very short period (<5 h per day), sleeping for a short period (5–7 h per day), sleeping for a normal period (7–9 h per day), and sleeping for a long period (≥9 h per day). These cutoffs are based on evidence-based recommendations from the National Sleep Foundation, establishing 7–9 h as the optimal range for adolescent health and cognitive function. Sleep duration was measured by asking participants, “During the past 7 days, what time did you usually go to bed, and what time did you wake up?” The study analyzed data based on average sleep duration [29].

#### 2.3.2. Dietary Health

Dietary intake was assessed using the KYRBS questionnaire on adolescent health behaviors and dietary habits. Breakfast habits were evaluated based on the following question: “In the past 7 days, how many days did you eat breakfast?” Participants reporting 7 days were classified as meeting the breakfast intake recommendations according to dietary guidelines for health [31,32]. Caffeine consumption classification is established based on adolescent-specific metabolic characteristics and physiological safety thresholds. Due to the incomplete maturation of hepatic metabolic enzyme activity in adolescents, caffeine has a longer half-life compared to adults, and brain adenosine receptors are more sensitive to caffeine; therefore, 125 mg/day (2.5 mg/kg body weight, based on a 50 kg standard) is established as the safety limit. Each serving of a high-caffeine beverage contains 58.1 mg of caffeine, and consuming a high-caffeine beverage 3 times daily (174.3 mg) significantly exceeds the safety threshold, representing a clear elevated risk level. Based on this, participants were categorized into low-frequency intake group (<3 times/day) and high-frequency intake group (≥3 times/day) [33].

#### 2.3.3. Physical Activity

The KYRBS includes six questions assessing vigorous, moderate, and light-intensity physical activity, adapted from the International Physical Activity Questionnaire (IPAQ) [34]. Based on ACSM guidelines and IPAQ classification criteria, participants were categorized into “Inadequate” or “Adequate” groups if they met any one of the following thresholds: ≥3 sessions/week of vigorous PA (20 min/session); ≥5 sessions/week of moderate-intensity PA (30 min/session); and ≥60 min/day of walking accumulated over 5 days/week. This classification system has been widely applied in both Korean and U.S. population studies [35].

#### 2.3.4. Sedentary Time

Sedentary behavior was assessed using American Academy of Pediatrics (AAP) screen time recommendations as the standard [36]. Participants provided self-reported data on the average time—measured in hours and minutes—they spent each day on screen-based activities, including watching television, playing computer games, and browsing the internet, during both weekdays and weekends. This data was then used to quantify screen-based sedentary behavior. According to AAP guidelines that establish health risk thresholds, sedentary activity levels were classified as low if screen time was less than 2 h per day, moderate if it ranged between 2 and 4 h per day, and high if it exceeded 4 h per day [37].

#### 2.3.5. Smoking

Smoking status was determined using two questions: ‘Have you smoked regular cigarettes in the past 6 months?’ and ‘Have you used any other types of cigarettes?’ Participants were classified as either smokers or non-smokers based on their responses [38].

#### 2.3.6. Hazardous Drinking

Alcohol consumption was assessed with the question: “On average, how much alcohol did you consume during each drinking occasion in the past month?” For the classification of risky drinking behavior, gender-differentiated alcohol consumption criteria were applied. Specifically, men who consumed 8 or more bottles of beer or more than 5 shots of soju in a single session and women who consumed more than 1 bottle of beer or 3 shots of soju per session were classified as risky drinkers [39].

### 2.4. Covariates

This study considered several confounding factors related to suicide risk, including bullying exposure, perceived health, economic status, and academic achievement. Bullying exposure was measured by asking the following question: ‘Have you ever required hospitalization due to violence—whether physical, psychological, or otherwise—inflicted by peers, superiors, or adults?’ [40]. Participants reporting multiple incidents were classified as having experienced bullying. Perceived health, economic status, and academic performance were initially categorized into five levels: ‘high,’ ‘upper-middle,’ ‘middle,’ ‘lower-middle,’ and ‘low,’ but for analysis, these levels were consolidated into three groups: ‘high,’ ‘middle,’ and ‘low.’

### 2.5. BMI

BMI is a widely used measure for classifying individuals as underweight, overweight, or obese. In 2000, the International Obesity Task Force (IOTF) developed BMI reference curves for children aged 2 to 18 years, using data from six international datasets [41], now known as the IOTF standards. For Asian pediatric populations, a BMI of 18.5 kg/m^2^ marks the threshold for underweight, while 23 kg/m^2^ and 27 kg/m^2^ serve as the recognized cutoffs for overweight and obesity, respectively [41].

$$\mathrm{BMI}=\frac{\mathrm{weight}\;(\mathrm{kg})}{{\mathrm{height}\;(m)}^{2}}$$

### 2.6. Data Analysis

All statistical analyses accounted for the complex survey design (stratification, clustering, and weighting) following KCDA guidelines. A non-parametric chi-square test was initially performed, followed by logistic regression to estimate odds ratios (OR) and their 95% confidence intervals (CIs). Multicollinearity assessment revealed VIF values of 1.012–1.220 and tolerance values of 0.820–0.988 for all variables, confirming no multicollinearity issues. Power analysis using G*Power 3.1.9.7 showed that with N = 38,412, α = 0.05, and medium effect size (OR = 2.0), the achieved statistical power was 1.00. Three logistic regression models were constructed: Model 1 was unadjusted; Model 2 was adjusted for gender, grade, academic performance, economic status, experience of bullying, and self-reported health; and Model 3 incorporated subgroup analyses based on BMI to investigate relationships within different population groups. Statistical significance was determined at a *p*-value of less than 0.05. Data analysis was carried out using SPSS version 25.0.

## 3. Results

### 3.1. Participant Characteristics

Table 1 presents an overview of the demographic and lifestyle characteristics of the study participants. The sample included 38,412 individuals, with 51.9% being male and 55.8% attending middle school. Concerning the subjective health assessment, 66.1% of participants rated their physical health as high level, while 2% reported having experienced violent victimization. In terms of dietary habits, 72.1% exhibited unhealthy breakfast patterns, and 20.8% reported frequent coffee consumption. When assessing physical activity, 14.2% had inadequate physical activity levels. With regard to sedentary behavior, only 6% of participants reported a low to moderate amount of sedentary time, whereas 94% displayed a high amount of sedentary time. Furthermore, 8.1% of participants reported sleeping less than five hours per night. As for health risk behaviors, 9.4% of participants reported smoking, and 5.1% were categorized as engaging in hazardous drinking. Finally, with respect to suicide-related behaviors, 13.3% of adolescents reported having suicidal ideation in the past year, 4% had made suicide plans, and 2.2% had attempted suicide at least once.

### 3.2. Association Between Lifestyle Factors and Adolescent Suicide Risk

Table 2 demonstrates the association between lifestyle factors and adolescent suicide risk. Eating an unhealthy breakfast was significantly associated with an increased risk of suicidal ideation (OR = 1.323, 95% CI [1.088, 1.610]). Frequent coffee consumption was also linked to a higher suicide risk. In Model 1, frequent coffee consumption was associated with suicidal ideation (OR = 1.381, 95% CI [1.191, 1.602]), suicide planning (OR = 1.563, 95% CI [1.247, 1.960]), and suicide attempts (OR = 1.733, 95% CI [1.316, 2.283]). In Model 2, the association between frequent coffee consumption and suicidal ideation (OR = 1.347, 95% CI [1.153, 1.573]), suicide planning (OR = 1.492, 95% CI [1.183, 1.882]), and suicide attempts (OR = 1.706, 95% CI [1.282, 2.271]) remained significant. Non-smoking behavior was significantly associated with a lower risk of suicide. In Model 1, those who did not smoke exhibited lower risks of suicidal ideation (OR = 0.737, 95% CI [0.631, 0.861]), suicide planning (OR = 0.712, 95% CI [0.561, 0.904]), and suicide attempts (OR = 0.583, 95% CI [0.436, 0.780]). After adjusting for confounding variables, the association remained significant, with those who did not smoke showing reduced risks of suicidal ideation (OR = 0.672, 95% CI [0.567, 0.796]), suicide planning (OR = 0.722, 95% CI [0.559, 0.932]), and suicide attempts (OR = 0.552, 95% CI [0.402, 0.757]). A sleep duration of less than 5 h was associated with a higher risk of suicide. In Model 1, a significant association with suicidal ideation was observed (OR = 1.736, 95% CI [1.163, 2.592]). In Model 2, insufficient sleep was significantly related to suicidal ideation (OR = 2.129, 95% CI [1.391, 3.259]), suicide planning (OR = 2.305, 95% CI [1.152, 4.611]), and suicide attempts (OR = 2.415, 95% CI [1.025, 5.690]). Hazardous drinking behavior was significantly associated with a higher risk of suicide planning (OR = 1.290, 95% CI [1.017, 1.637]), and this association remained significant in Model 2 (OR = 1.300, 95% CI [1.010, 1.673]). However, physical activity and sedentary time were not significantly associated with suicidal risk behaviors.

### 3.3. Subgroup Analysis of Adolescents

Table 3 presents the subgroup analysis for underweight participants, showing that frequent coffee consumption was associated with an increased risk of suicide. In the unadjusted model, significant associations were observed with suicidal ideation (OR = 1.499, 95% CI [1.065, 2.109]), suicide planning (OR = 1.695, 95% CI [1.003, 2.867]), and suicide attempts (OR = 2.167, 95% CI [1.180, 3.982]). After adjusting for confounders, the association with suicidal ideation was no longer significant, but the associations with suicide planning (OR = 1.695, 95% CI [1.003, 2.867]) and suicide attempts (OR = 2.216, 95% CI [1.153, 4.258]) remained significant. Among underweight participants, sleeping less than 5 h per night was significantly associated with suicidal ideation (OR = 2.391, 95% CI [1.035, 5.525]), and this association persisted after adjusting for covariates (OR = 3.416, 95% CI [1.403, 8.317]). Non-smoking behavior was linked to a lower risk of suicide attempts (OR = 0.451, 95% CI [0.236, 0.864]). No significant associations were identified between breakfast habits, physical activity, sedentary behavior, or hazardous drinking behavior and suicide risk among underweight participants.

Table 4 presents the results from the subgroup analysis of overweight adolescents, highlighting the association between frequent coffee consumption and suicide risk. In Model 1, frequent coffee consumption was significantly associated with increased risks of suicide attempts (OR = 1.958, 95% CI [1.024, 3.742]) and suicide planning (OR = 1.939, 95% CI [1.133, 3.020]). These associations remained significant in Model 2, with frequent coffee consumption linked to both suicide attempts (OR = 2.406, 95% CI [1.211, 4.783]) and suicide planning (OR = 1.939, 95% CI [1.163, 3.233]). For health risk behaviors, those who did not smoke had a significantly lower risk of suicidal ideation compared to smokers in the unadjusted model (OR = 0.706, 95% CI [0.507, 0.983]). After controlling for confounders, non-smoking behavior remained significantly associated with reduced risks of suicidal ideation (OR = 0.566, 95% CI [0.393, 0.815]) and suicide planning (OR = 0.528, 95% CI [0.299, 0.930]). Hazardous drinking behavior was also significantly associated with a higher risk of suicide attempts in overweight adolescents (*p* = 0.021, OR = 2.277, 95% CI [1.132, 4.580]). No significant associations were observed between breakfast habits, physical activity, sedentary behavior, or sleep and suicidal risk behaviors in this group.

Table 5 presents the subgroup analysis results for obese participants. In the adjusted model, non-smoking among obese adolescents was significantly associated with a reduced risk of suicidal ideation (OR = 0.514, 95% CI [0.297, 0.887]). Furthermore, frequent coffee consumption was found to significantly increase the risk of suicide attempts (*p* = 0.039, OR = 2.492, 95% CI [1.048, 5.925]). Among all variables assessed, only smoking and coffee consumption demonstrated statistically significant associations with suicidal behaviors among obese adolescents.

## 4. Discussion

This study, using data from the 2022 KYRBS conducted by the KDCA, analyzed the overall impact of various lifestyle factors on suicide-related behaviors. First, our analysis highlighted an increased risk of suicide-related behaviors associated with unhealthy breakfast habits, frequent coffee consumption, hazardous drinking, smoking, and sleep durations of less than five hours. Second, no significant associations were found between physical activity or sedentary time and adolescent suicide-related behaviors. Notably, significant differences emerged in how lifestyle factors influenced suicide-related behaviors across different BMI categories. Specifically, a sleep duration of less than 5 h had a substantial impact on suicide-related behaviors in underweight adolescents, while smoking was a critical determinant of suicide behaviors in overweight and obese adolescents.

Our study reveals that a sleep duration of less than 5 h significantly increases the likelihood of suicide-related behaviors in adolescents, corroborating previous findings [42]. Similarly, the U.S. Youth Risk Behavior Survey has demonstrated that adolescents sleeping less than eight hours face a heightened risk of engaging in suicidal behaviors [43]. Notably, we identified insufficient sleep as a key determinant of suicide risk among underweight adolescents, potentially linking low body weight, sleep disturbances, and negative emotional states [44]. Being underweight during adolescence may stem from excessive weight loss during puberty or underlying mental health conditions. Additionally, our findings suggest that underweight adolescents are especially vulnerable to the adverse effects of sleep deprivation, which may contribute to higher mortality rates and a reduced life expectancy [45]. Major risk factors include eating disorders, neurological conditions, sleep pattern disruptions, and depression [46]. The combined presence of underweight status and sleep deprivation should be regarded as a critical indicator of suicidal ideation.

Furthermore, our examination of caffeine consumption patterns revealed a dose-dependent relationship with suicide-related behaviors. Excessive caffeine intake, particularly when not adjusted for confounding factors, significantly increases the risk of suicidal ideation [47]. A noteworthy finding of this study is that not smoking is a protective factor against suicidal behaviors. This could be explained by the role smoking plays in increasing one’s suicide risk, potentially through exacerbating impulsivity [48,49,50,51,52,53,54,55,56,57]. From a physiological standpoint, smoking likely enhances impulsive behaviors, raising suicide risk via pathways involving serotonin regulation [51], MAOA gene variants [52], and nicotine dependence [49]. Although both caffeine and nicotine are classified as stimulants, their different associations with suicidal behaviors can be explained by their distinct neurophysiological mechanisms. Caffeine primarily operates through the antagonism of adenosine receptors, leading to increased dopaminergic activity and enhanced arousal [53]. This mechanism, while stimulating, does not directly implicate the same impulsivity pathways associated with suicidal behaviors [48]. In contrast, nicotine’s action on nicotinic acetylcholine receptors has more direct effects on serotonergic and dopaminergic transmission patterns associated with mood regulation and impulsivity [54,55]. Additionally, nicotine produces a more pronounced withdrawal syndrome characterized by irritability, anxiety, and depressed mood—psychological states closely linked to suicidal ideation [56]. Furthermore, our study revealed that the combination of smoking and obesity or overweight status significantly increases the likelihood of suicidal behaviors among adolescents. This finding aligns with previous adult research, which has demonstrated a strong link between obesity, smoking, and increased psychological burden [57].

Furthermore, our study indicates that adolescents who engage in hazardous drinking behavior have an increased likelihood of engaging in suicide-related behaviors. The mechanisms linking obesity to suicidality in recent years remain incompletely understood. Studies of U.S. adolescents and emerging adults have found that alcohol consumption mediates the relationship between impulsivity and self-harm [58]. Adolescent alcohol misuse serves as a significant predictor of self-harm and suicidality in early adulthood [59]. Chronic alcohol use disrupts white and gray matter development in the brain, impairs normal function in cortical and hippocampal regions, activates non-neuronal cells, and triggers pro-inflammatory signaling, leading to alcohol-induced cognitive deficits [60]. Pharmacologically, alcohol is a central nervous system (CNS) depressant [61]. From a neurobiological perspective, long-term alcohol consumption disrupts serotonergic function, which plays a critical role in mood regulation and impulse control [62]. Unlike caffeine and nicotine, which primarily act as stimulants, alcohol consumption inhibits excitatory neurotransmission (especially glutamate) and enhances inhibitory neurotransmission (mainly GABA), resulting in overall CNS depression [55,63]. Alcohol directly impairs cognitive function and may weaken an individual’s ability to generate alternative solutions, thereby increasing the likelihood of considering suicide as a viable option during emotional crises [64]. Therefore, broader environmental and social factors are the primary drivers of suicide risk, with alcohol amplifying existing vulnerabilities. Heavy alcohol use among obese adolescents may act as a screening marker for suicide risk. A U.S.-based study revealed that solitary drinking is associated with a higher BMI, poorer sleep quality, and increased sugar intake [65]. These findings suggest that the influence of drinking patterns on suicidality requires further longitudinal investigation. Previous research has highlighted unique associations between obesity-related high-risk drinking and two key components of decision making [66]. The physiological interplay between adipose tissue in obesity, a heightened suicide risk, and other endogenous environmental factors is complex.

Our study further analyzes the risk factors for suicidal behavior among obese, overweight, and underweight adolescents. Previous studies have reported associations between weight abnormalities and suicide [8]. Related research indicates that many mental disorders are closely associated with adolescent obesity, including depression, bipolar disorder, and psychiatric disorders [67]. Meanwhile, factors such as psychosocial stigma and bullying are also associated with both suicidal behavior and obesity [68]. A self-perceived abnormal weight during adolescence is closely associated with negative mental health outcomes, including suicidal behavior, which is consistent with the results shown in this study [46]. However, recent interdisciplinary research has proposed a new theoretical perspective, suggesting that suicide risk among obese and overweight adolescents stems more from deteriorating interpersonal relationships and social disconnection rather than purely psychological distress [13]. We need to reexamine the mechanisms linking weight abnormalities and suicidal behavior. Weight-related social stigmatization leads to peer rejection and social isolation, causing adolescents to lose important social support [69]. The deterioration of these interpersonal relationships may more directly affect adolescents’ mental health and suicide risk than the weight problems themselves.

Contrary to our expectations, our analysis revealed no significant associations between physical activity or sedentary time and suicidal risk behaviors. This divergence from the established literature warrants careful consideration. We propose several explanations for these results. First, the relationship between physical activity and mental health may be curvilinear rather than linear, with both insufficient and excessive exercise potentially yielding negative psychological outcomes [70,71]. The social context aspects of physical activity may have a greater impact [72]. Regarding sedentary behavior, our findings may reflect that the psychological impact of screen time on adolescents might be more nuanced than previously understood. Recent evidence suggests that content and context determine whether screen time is harmful or beneficial [73].

This study has several limitations. First, our cross-sectional design cannot capture the dynamic nature of social relationships and their evolving impact on suicide risk. Second, causal relationships between lifestyle factors and suicide-related outcomes cannot be definitively inferred. Third, adolescent self-reports may be affected by social desirability bias and recall errors. Fourth, excluding participants with incomplete data may introduce selection bias and limit the study’s generalizability. Fifth, although our subgroup analysis provided valuable insights, the relatively small sample sizes in the overweight and obese groups resulted in wider confidence intervals, reducing its statistical power. Finally, our findings specifically pertain to suicidal ideation, planning, and attempts, rather than suicide mortality.

## 5. Conclusions

This study found that a sleep duration of less than 5 h, unhealthy breakfast habits, frequent coffee consumption, hazardous drinking, and smoking significantly increase suicide risk, while no significant associations were found between physical activity or sedentary time and suicidal behaviors. Sleep deprivation (sleeping <5 h) is a key determinant of suicide risk among underweight adolescents. The combination of smoking with a higher Body Mass Index may contribute to an elevated risk. Attention should be paid to the interplay between smoking, unhealthy dietary patterns, sleep, and hazardous drinking behavior and BMI for the early detection and prevention of adolescent suicide. Schools should simultaneously assess BMI, sleep patterns, smoking status, and substance use behaviors to identify high-risk students early. Healthcare systems should incorporate BMI-specific risk assessments into suicide prevention protocols. Future research requires longitudinal studies to establish causality and identify critical intervention periods.

## Figures and Tables

**Table 1:** Characteristics of study subjects (N = 38,412).

Variables	Categories	Total (%)
Gender	Male	19,946 (51.9)
	Female	18,466 (48.1)
Grade	Middle School	21,442 (55.8)
	High School	16,970 (44.2)
Economic	Low Level	3978 (10.4)
	Middle Level	17,806 (46.4)
	High Level	16,628 (43.3)
Subjective health assessment	Low Level	13,014 (33.9)
	High Level	25,398 (66.1)
Achievement	Low Level	11,210 (29.2)
	Middle Level	11,584 (30.2)
	High Level	15,618 (40.7)
Violent victimization	No	37,651 (98)
	Yes	761 (2)
Healthy diet		
Breakfast	Unhealthy	27,706 (72.1)
	Healthy	10,706 (27.9)
Coffee	Frequent	7998 (20.8)
	Infrequent	30,414 (79.2)
Physical activity	Inadequate	5455 (14.2)
	Adequate	32,957 (85.8)
Sedentary time	Low Level	524 (1.4)
	Middle Level	1765 (4.6)
	High Level	36,123 (94)
Sleep	<5 h	3115 (8.1)
	5–7 h	18,381 (47.9)
	7–9 h	14,334 (37.3)
	≥9 h	2582 (6.7)
Health risk behaviors		
Cigarettes	No	34,801 (90.6)
	Yes	3111 (9.4)
Hazardous drinking	Yes	1942 (5.1)
	No	2836 (7.4)
Suicidal ideation	No	33,296 (86.7)
	Yes	5116 (13.3)
Suicide planning	No	36,867 (96)
	Yes	1545 (4)
Suicide attempt	No	37,549 (97.8)
	Yes	863 (2.2)
Body Mass Index	Underweight	9029 (23.5)
	Normal weight	18,283 (47.5)
	Overweight	7729 (20.2)
	Obesity	3371 (8.8)

**Table 2:** Odds ratios and 95% confidence intervals of lifestyle factors for suicidal ideation, planning, and attempts among all adolescents (N = 38,412).

	Suicidal Ideation		Suicide Planning		Suicide Attempt	
	Crude	Adjusted ^a^	Crude	Adjusted ^a^	Crude	Adjusted ^a^
	Odds Ratio (95% Confidence Intervals)					
Healthy diet						
Breakfast						
Unhealthy	1.323 (1.088–1.610) **	1.067 (0.868–1.312)	1.060 (0.789–1.424)	0.889 (0.654–1.209)	1.249 (0.853–1.828)	0.964 (0.647–1.436)
Healthy	1 (Ref.)	1 (Ref.)	1 (Ref.)	1 (Ref.)	1 (Ref.)	1 (Ref.)
Coffee						
Frequent	1.381 (1.191–1.602) ***	1.347 (1.153–1.573) ***	1.563 (1.247–1.960) ***	1.492 (1.183–1.882) **	1.733 (1.316–2.283) ***	1.706 (1.282–2.271) ***
Infrequent	1 (Ref.)	1 (Ref.)	1 (Ref.)	1 (Ref.)	1 (Ref.)	1 (Ref.)
Physical Activity						
Inadequate	1.012 (0.802–1.277)	1.032 (0.809–1.316)	0.685 (0.450–1.044)	0.714 (0.465–1.096)	0.700 (0.416–1.179)	0.737 (0.432–1.257)
Adequate	1 (Ref.)	1 (Ref.)	1 (Ref.)	1 (Ref.)	1 (Ref.)	1 (Ref.)
Sedentary Time						
Low Level	1.048 (0.638–1.720)	1.045 (0.613–1.779)	1.100 (0.525–2.305)	1.002 (0.457–2.194)	1.012 (0.403–2.540)	0.875 (0.317–2.415)
Middle Level	0.822 (0.575–1.175)	0.858 (0.592–1.245)	1.175 (0.720–1.917)	1.206 (0.728–1.997)	1.306 (0.741–2.302)	1.473 (0.815–2.662)
High Level	1 (Ref.)	1 (Ref.)	1 (Ref.)	1 (Ref.)	1 (Ref.)	1 (Ref.)
Sleep						
<5 h	1.736 (1.163–2.592) **	2.129 (1.391–3.259) **	1.890 (0.967–3.694)	2.305 (1.152–4.611) **	2.000 (0.878–4.556) *	2.415 (1.025–5.690) *
5–7 h	0.876 (0.607–1.264)	1.202 (0.816–1.772)	1.106 (0.589–2.079)	1.569 (0.819–3.005)	1.008 (0.461–2.208)	1.527 (0.678–3.437)
7–9 h	0.784 (0.535–1.148)	1.002 (0.671–1.494)	1.022 (0.532–1.963)	1.334 (0.684–2.604)	1.148 (0.513–2.565)	1.619 (0.706–3.711)
≥9	1 (Ref.)	1 (Ref.)	1 (Ref.)	1 (Ref.)	1 (Ref.)	1 (Ref.)
Health Risk Behaviors						
Cigarettes						
No	0.737 (0.631–0.861) ***	0.672 (0.567–0.796) ***	0.712 (0.561–0.904) **	0.722 (0.559–0.932) **	0.583 (0.436–0.780) ***	0.552 (0.402–0.757) ***
Yes	1 (Ref.)	1 (Ref.)	1 (Ref.)	1 (Ref.)	1 (Ref.)	1 (Ref.)
Hazardous Drinking						
Yes	0.973 (0.833–1.137)	0.975 (0.825–1.152)	1.290 (1.017–1.637) *	1.300 (1.010–1.673) *	1.269 (0.948–1.699)	1.171 (0.857–1.600)
No	1 (Ref.)	1 (Ref.)	1 (Ref.)	1 (Ref.)	1 (Ref.)	1 (Ref.)
R^2^	0.231	0.453	0.058	0.125	0.034	0.116

^a^ Adjusted for gender, grade, academic achievement, economic status, violent victimization, and self-perceived health. * *p* < 0.05. ** *p* < 0.01. *** *p* < 0.001.

**Table 3:** Odds ratios and 95% confidence intervals of lifestyle factors for suicidal ideation, planning, and attempts among underweight adolescents (N = 9029).

	Suicidal Ideation		Suicide Planning		Suicide Attempt	
	Crude	Adjusted ^a^	Crude	Adjusted ^a^	Crude	Adjusted ^a^
	Odds Ratio (95% Confidence Intervals)					
Healthy Diet						
Breakfast						
Unhealthy	1.488 (0.934–2.372)	1.383 (0.848–2.255)	1.000 (0.499–2.005)	1.000 (0.499–2.005)	1.107 (0.473–2.588)	0.992 (0.403–2.441)
Healthy	1 (Ref.)	1 (Ref.)	1 (Ref.)	1 (Ref.)	1 (Ref.)	1 (Ref.)
Coffee						
Frequent	1.499 (1.065–2.109) *	1.419 (0.987–2.040)	1.695 (1.003–2.867) *	1.695 (1.003–2.867) *	2.167 (1.180–3.982) *	2.216 (1.153–4.258) *
Infrequent	1 (Ref.)	1 (Ref.)	1 (Ref.)	1 (Ref.)	1 (Ref.)	1 (Ref.)
Physical Activity						
Inadequate	0.750 (0.435–1.292)	0.785 (0.445–1.384)	0.367 (0.110–1.220)	0.367 (0.110–1.220)	0.596 (0.177–2.010)	0.609 (0.165–2.243)
Adequate	1 (Ref.)	1 (Ref.)	1 (Ref.)	1 (Ref.)	1 (Ref.)	1 (Ref.)
Sedentary Time						
Low Level	1.296 (0.308–5.453)	1.000 (0.228–4.393)	1.415 (0.163–12.291)	1.415 (0.163–12.291)	1.873 (0.212–16.550)	1.820 (0.156–21.215)
Middle Level	0.896 (0.410–1.959)	0.996 (0.441–2.246)	0.916 (0.261–3.213)	0.916 (0.261–3.213)	0.954 (0.214–4.250)	1.134 (0.241–5.346)
High Level	1 (Ref.)	1 (Ref.)	1 (Ref.)	1 (Ref.)	1 (Ref.)	1 (Ref.)
Sleep						
<5 h	2.391 (1.035–5.525) *	3.416 (1.403–8.317) **	2.179 (0.534–8.890)	2.179 (0.534–8.890)	2.825 (0.582–13.711)	3.093 (0.590–16.215)
5–7 h	1.193 (0.565–2.517)	1.718 (0.783–3.772)	1.786 (0.503–6.344)	1.786 (0.503–6.344)	1.008 (0.222–4.574)	1.268 (0.270–5.953)
7–9 h	0.900 (0.413–1.961)	1.215 (0.539–2.738)	1.250 (0.334–4.683)	1.250 (0.334–4.683)	0.878 (0.182–4.234)	1.113 (0.222–5.566)
≥9	1 (Ref.)	1 (Ref.)	1 (Ref.)	1 (Ref.)	1 (Ref.)	1 (Ref.)
Health Risk Behaviors						
Cigarettes						
No	0.699 (0.488–1.003)	0.771 (0.522–1.136)	0.982 (0.542–1.781)	0.982 (0.542–1.781)	0.451 (0.236–0.864) *	0.490 (0.237–1.014)
Yes	1 (Ref.)	1 (Ref.)	1 (Ref.)	1 (Ref.)	1 (Ref.)	1 (Ref.)
Hazardous Drinking						
Yes	0.843 (0.586–1.214)	0.841 (0.569–1.243)	0.836 (0.461–1.518)	0.836 (0.461–1.518)	0.760 (0.387–1.493)	0.657 (0.308–1.401)
No	1 (Ref.)	1 (Ref.)	1 (Ref.)	1 (Ref.)	1 (Ref.)	1 (Ref.)
R^2^	0.257	0.296	0.076	0.160	0.067	0.201

^a^ Adjusted for gender, grade, academic achievement, economic status, violent victimization, and self-perceived health. * *p* < 0.05. ** *p* < 0.01.

**Table 4:** Odds ratios and 95% confidence intervals of lifestyle factors for suicidal ideation, planning, and attempts among overweight adolescents (N = 7729).

	Suicidal Ideation		Suicide Planning		Suicide Attempt	
	Crude	Adjusted ^a^	Crude	Adjusted ^a^	Crude	Adjusted ^a^
	Odds Ratio (95% Confidence Intervals)					
Healthy Diet						
Breakfast						
Unhealthy	0.989 (0.670–1.459)	0.768 (0.508–1.164)	0.693 (0.393–1.222)	0.534 (0.290–0.983)	0.451 (0.226–0.901)	0.307 (0.140–0.672)
Healthy	1 (Ref.)	1 (Ref.)	1 (Ref.)	1 (Ref.)	1 (Ref.)	1 (Ref.)
Coffee						
Frequent	1.198 (0.871–1.646)	1.259 (0.900–1.759)	1.850 (1.133–3.020) *	1.939 (1.163–3.233) *	1.958 (1.024–3.742) *	2.406 (1.211–4.783) **
Infrequent	1 (Ref.)	1 (Ref.)	1 (Ref.)	1 (Ref.)	1 (Ref.)	1 (Ref.)
Physical Activity						
Inadequate	1.440 (0.903–2.298)	1.391 (0.845–2.290)	1.007 (0.444–2.286)	0.925 (0.385–2.220)	0.506 (0.119–2.157)	0.550 (0.124–2.447)
Adequate	1 (Ref.)	1 (Ref.)	1 (Ref.)	1 (Ref.)	1 (Ref.)	1 (Ref.)
Sedentary Time						
Low Level	1.234 (0.478–3.185)	1.113 (0.373–3.319)	1.787 (0.506–6.309)	1.434 (0.325–6.324)	-------------------	------------------
Middle Level	0.919 (0.437–1.931)	0.899 (0.417–1.940)	2.201 (0.933–5.195)	2.431 (0.982–6.019)	2.317 (0.846–6.348)	2.802 (0.958–8.196)
High Level	1 (Ref.)	1 (Ref.)	1 (Ref.)	1 (Ref.)	1 (Ref.)	1 (Ref.)
Sleep						
<5 h	2.397 (0.921–6.241)	2.593 (0.937–7.172)	4.487 (0.564–35.716)	5.655 (0.649–49.248)	0.282 (0.076–1.042)	0.216 (0.040–1.162)
5–7 h	01.252 (0.513–3.055)	1.822 (0.711–4.672)	2.194 (0.290–16.585)	4.129 (0.500–34.065)	0.282 (0.076–1.042)	0.355 (0.088–1.430)
7–9 h	1.121 (0.446–2.821)	1.479 (0.561–3.902)	2.853 (0.369–22.048)	5.242 (0.618–44.432)	0.595 (0.159–2.222)	0.694 (0.171–2.820)
≥9	1 (Ref.)	1 (Ref.)	1 (Ref.)	1 (Ref.)	1 (Ref.)	1 (Ref.)
Health Risk Behaviors						
Cigarettes						
No	0.706 (0.507–0.983) *	0.566 (0.393–0.815) **	0.644 (0.384–1.079)	0.528 (0.299–0.930) *	0.840 (0.427–1.651)	0.522 (0.239–1.138)
Yes	1 (Ref.)	1 (Ref.)	1 (Ref.)	1 (Ref.)	1 (Ref.)	1 (Ref.)
Hazardous Drinking						
Yes	1.076 (0.772–1.500)	1.045 (0.731–1.495)	1.215 (0.721–2.047)	1.138 (0.645–2.007)	2.277 (1.132–4.580) *	2.098 (0.974–4.518)
No	1 (Ref.)	1 (Ref.)	1 (Ref.)	1 (Ref.)	1 (Ref.)	1 (Ref.)
R^2^	0.183	0.638	0.030	0.093	0.098	1.156

^a^ Adjusted for gender, grade, academic achievement, economic status, violent victimization, and self-perceived health. * *p* < 0.05. ** *p* < 0.01.

**Table 5:** Odds ratios and 95% confidence intervals of lifestyle factors for suicidal ideation, planning, and attempts among obese adolescents (N = 3371).

	Suicidal Ideation		Suicide Planning		Suicide Attempt	
	Crude	Adjusted ^a^	Crude	Adjusted ^a^	Crude	Adjusted ^a^
	Odds Ratio (95% Confidence Intervals)					
Healthy Diet						
Breakfast						
Unhealthy	1.507 (0.793–2.864)	1.254 (0.633–2.487)	2.265 (0.783–6.551)	1.843 (0.607–5.598)	0.675 (0.273–1.670)	0.477 (0.175–1.302)
Healthy	1 (Ref.)	1 (Ref.)	1 (Ref.)	1 (Ref.)	1 (Ref.)	1 (Ref.)
Coffee						
Frequent	1.337 (0.827–2.161)	1.348 (0.803–2.261)	0.998 (0.502–1.983)	0.898 (0.426–1.892)	2.094 (0.948–4.629)	2.492 (1.048–5.925) *
Infrequent	1 (Ref.)	1 (Ref.)	1 (Ref.)	1 (Ref.)	1 (Ref.)	1 (Ref.)
Physical Activity						
Inadequate	1.348 (0.639–2.843)	1.485 (0.655–3.367)	0.783 (0.230–2.664)	0.910 (0.245–3.377)	0.361 (0.047–2.749)	0.362 (0.042–3.119)
Adequate	1 (Ref.)	1 (Ref.)	1 (Ref.)	1 (Ref.)	1 (Ref.)	1 (Ref.)
Sedentary Time						
Low Level	1.620 (0.551–4.762)	1.946 (0.614–6.166)	1.193 (0.256–5.556)	1.303 (0.236–7.182)	2.874 (0.734–11.256)	3.847 (0.831–17.813)
Middle Level	0.510 (0.114–2.284)	0.422 (0.089–2.006)	1.108 (0.239–5.132)	1.222 (0.246–6.073)	0.855 (0.104–7.033)	0.581 (0.059–5.765)
High Level	1 (Ref.)	1 (Ref.)	1 (Ref.)	1 (Ref.)	1 (Ref.)	1 (Ref.)
Sleep						
<5 h	2.446 (0.482–12.413)	3.242 (0.550–19.098)	------------------	------------------	------------------	------------------
5–7 h	1.256 (0.274–5.753)	1.825 (0.347–9.613)	------------------	------------------	------------------	------------------
7–9 h	1.574 (0.333–7.445)	1.912 (0.352–10.385)	------------------	------------------	------------------	------------------
≥9	1 (Ref.)	1 (Ref.)	1 (Ref.)	1 (Ref.)	1 (Ref.)	1 (Ref.)
Health Risk Behaviors						
Cigarettes						
No	0.655 (0.400–1.071)	0.514 (0.297–0.887) *	0.893 (0.448–1.782)	0.728 (0.337–1.573)	0.797 (0.354–1.793)	0.764 (0.302–1.932)
Yes	1 (Ref.)	1 (Ref.)	1 (Ref.)	1 (Ref.)	1 (Ref.)	1 (Ref.)
Hazardous Drinking						
Yes	0.762 (0.459–1.2660)	0.731 (0.412–1.295)	1.385 (0.687–2.793)	1.102 (0.505–2.406)	1.694 (0.730–3.929)	1.686 (0.646–4.400)
No	1 (Ref.)	1 (Ref.)	1 (Ref.)	1 (Ref.)	1 (Ref.)	1 (Ref.)
R^2^	0.113	0.142	0.000	0.000	0.000	0.000

^a^ Adjusted for gender, grade, academic achievement, economic status, violent victimization, and self-perceived health. * *p* < 0.05.

## Data Availability

This study utilized data from the KYRBS conducted by the KDCA. This survey is an open-access dataset, and researchers can apply for access through the official KDCA website: www.kdca.go.kr (accessed on 15 March 2024).
